# Supplementary material for: Analysis of long and short enhancers in melanoma cell states
Source: eLife. 2021 Dec 7;10:e71735. doi: 10.7554/eLife.71735 (PMC8691835; doi:10.7554/eLife.71735)
Supplement: Transparent reporting form [file elife-71735-transrepform1.docx]

***eLife’s* transparent reporting form**

We encourage authors to provide detailed information *within their submission* to facilitate the interpretation and replication of experiments. Authors can upload supporting documentation to indicate the use of appropriate reporting guidelines for health-related research (see [EQUATOR Network](http://www.equator-network.org/%20)), life science research (see the [BioSharing Information Resource](https://biosharing.org/)), or the [ARRIVE guidelines](http://www.plosbiology.org/article/info:doi/10.1371/journal.pbio.1000412) for reporting work involving animal research. Where applicable, authors should refer to any relevant reporting standards documents in this form.

If you have any questions, please consult our Journal Policies and/or contact us: [editorial@elifesciences.org](mailto:editorial@elifesciences.org).

**Sample-size estimation**

- You should state whether an appropriate sample size was computed when the study was being designed
- You should state the statistical method of sample size computation and any required assumptions
- If no explicit power analysis was used, you should describe how you decided what sample (replicate) size (number) to use

Please outline where this information can be found within the submission (e.g., sections or figure legends), or explain why this information doesn’t apply to your submission:

No power analysis was performed to estimate appropriate sample size. Each MPRA assay contains thousands of individual barcode measurements and includes its own controls, making it a high-throughput screen even when the number of tested enhancers is small. Statistics were calculated within the sample and not across experiments. Ashuach et al. (Genome Biology, 2019) showed that increasing the number of barcodes has a similar effect as increasing the number of replicates, since both parameters increase the effective sample size. Furthermore, for most MPRA libraries, activity profile was confirmed by changing enhancer position or reporter type.

**Replicates**

- You should report how often each experiment was performed
- You should include a definition of biological versus technical replication
- The data obtained should be provided and sufficient information should be provided to indicate the number of independent biological and/or technical replicates
- If you encountered any outliers, you should describe how these were handled
- Criteria for exclusion/inclusion of data should be clearly stated
- High-throughput sequence data should be uploaded before submission, with a private link for reviewers provided (these are available from both GEO and ArrayExpress)

Please outline where this information can be found within the submission (e.g., sections or figure legends), or explain why this information doesn’t apply to your submission:

A biological replicate corresponds to an individual transfection, extraction and PCR amplification of a cell line. The number of replicates per sample is indicated in “Methods – MPRA assay”. The criteria for exclusion/inclusion of data can be found in “Methods – MPRA analysis – Sample exclusion”.

MPRA data generated for this study have been submitted to the NCBI Gene Expression Omnibus under accession number GSE 180879.

**Statistical reporting**

- Statistical analysis methods should be described and justified
- Raw data should be presented in figures whenever informative to do so (typically when N per group is less than 10)
- For each experiment, you should identify the statistical tests used, exact values of N, definitions of center, methods of multiple test correction, and dispersion and precision measures (e.g., mean, median, SD, SEM, confidence intervals; and, for the major substantive results, a measure of effect size (e.g., Pearson's r, Cohen's d)
- Report exact p-values wherever possible alongside the summary statistics and 95% confidence intervals. These should be reported for all key questions and not only when the p-value is less than 0.05.

Please outline where this information can be found within the submission (e.g., sections or figure legends), or explain why this information doesn’t apply to your submission:

p-value calculation for enhancer activity is described in “Methods – MPRA analysis – Estimation of enhancer activity”. Pearson and Spearman correlations are calculated with the cor() R function and the “corrplot” package was used for visualization and clustering. For Figure 5—figure supplement 1 and Figure 6— figure supplement 1, comparison between wild type and mutant group is done by performing a t-test with the stat_compare_means() function from the “ggpubr” R package.

(For large datasets, or papers with a very large number of statistical tests, you may upload a single table file with tests, Ns, etc., with reference to sections in the manuscript.)

**Group allocation**

- Indicate how samples were allocated into experimental groups (in the case of clinical studies, please specify allocation to treatment method); if randomization was used, please also state if restricted randomization was applied
- Indicate if masking was used during group allocation, data collection and/or data analysis

Please outline where this information can be found within the submission (e.g., sections or figure legends), or explain why this information doesn’t apply to your submission:

MM001 was selected for representing MEL subtype because is it the most melanocytic line we have available (Wouters et al. 2020). MM057, MM074 and MM087 were all the intermediate lines we had available during this study. MM029, MM047 and MM099 were all the MES lines we had available during this study.

**Additional data files (“source data”)**

- We encourage you to upload relevant additional data files, such as numerical data that are represented as a graph in a figure, or as a summary table
- Where provided, these should be in the most useful format, and they can be uploaded as “Source data” files linked to a main figure or table
- Include model definition files including the full list of parameters used
- Include code used for data analysis (e.g., R, MatLab)
- Avoid stating that data files are “available upon request”

Please indicate the figures or tables for which source data files have been provided:

The following additional data files are provided:
- CHEQ-seq_5p_H3K27ac_processed.txt: containing enhancer activity values for the CHEQ-seq 5’ H3K27ac library used in Figure 2e., Figure 3e., Figure 6b., Figure 2—figure supplement 1a.-b., Figure 3—figure supplement 1a.-b.
- CHEQ-seq_Intron_H3K27ac_processed.txt: containing enhancer activity values for the CHEQ-seq Intron H3K27ac library used in Figure 2, Figure 3, Figure 4a.-c., Figure 5a.-c., Figure 6a.-b.-c.-f., Figure 2—figure supplement 1b.-e., Figure 2—figure supplement 2, Figure 3—figure supplement 1b.-e., Figure 3—figure supplement 2, Figure 4—figure supplement 1.
- CHEQ-seq_ATAC_processed.txt: containing enhancer activity values for the CHEQ-seq ATAC library used in Figure 2, Figure 3, Figure 4d.-f., Figure 5a., Figure 6a.-b.-c., Figure 2—figure supplement 1d.-e., Figure 2—figure supplement 2, Figure 3—figure supplement 1d.-e., Figure 3—figure supplement 2, Figure 4—figure supplement 1.
- STARR-seq_ATAC_processed.txt: containing enhancer activity values for the STARR-seq ATAC library used in Figure 2f., Figure 3f., Figure 6b., Figure 2—figure supplement 1c.-d., Figure 3—figure supplement 1c.-d.
- CHEQ-seq_Enhancer_tiling_A_processed.txt: containing enhancer activity values for the CHEQ-seq enhancer tiling library A used in Figure 3c.-d., Figure 4g.-i., Figure 6a.-c.-e., Figure 3—figure supplement 2, Figure 4—figure supplement 1, Figure 5—figure supplement 1, Figure 6—figure supplement 1.

- CHEQ-seq_Enhancer_tiling_B_processed.txt: containing enhancer activity values for the CHEQ-seq enhancer tiling library B used in Figure 2c., Figure 3c.-d., Figure 4g.-i., Figure 5a., Figure 6a.-c.-e., Figure 2—figure supplement 2, Figure 3—figure supplement 2, Figure 4—figure supplement 1, Figure 5—figure supplement 1, Figure 6—figure supplement 1.
- CHEQ-seq_SOX10-KD_processed.txt: containing enhancer activity values for the CHEQ-seq SOX10-KD library used in Figure 5d., Figure 5—figure supplement 4.
- CHEQ-seq_SOX-MITF_combinations processed.txt: containing enhancer activity values for the CHEQ-seq SOX-MITF combinations library used in Figure 5e.
- DeepMEL2_Enhancer_tiling_A.txt: containing DeepMEL2_GABPA predictions scores for the enhancer tiling A library used in Figure 3c.-d., Figure 6c., Figure 3—figure supplement 2, Figure 4—figure supplement 1.
- DeepMEL2_Enhancer_tiling_B.txt: containing DeepMEL2_GABPA predictions scores for the enhancer tiling B library used in Figure 2c., Figure 3c.-d., Figure 5a., Figure 6c., Figure 2—figure supplement 2, Figure 3—figure supplement 2, Figure 4—figure supplement 1.
- DeepMEL2_SOX-MITF_combinations.txt: containing DeepMEL2_GABPA predictions scores for the SOX-MITF combinations sequences used in Figure 5—figure supplement 5.

- Luc_values.csv: containing luciferase assay values used in Figure 5—figure supplement 3 and Figure 6—figure supplement 3

The code used for enhancer-barcode assignment, read processing and analysis is available on GitHub: https://github.com/aertslab/Melanoma_MPRA_paper
